# Targeting hedgehog signaling in myelofibrosis and other hematologic malignancies

**DOI:** 10.1186/1756-8722-7-18

**Published:** 2014-03-05

**Authors:** Raoul Tibes, Ruben A Mesa

**Affiliations:** 1 Mayo Clinic Cancer Center, NCI Designated Comprehensive Cancer Center, 13400 E. Shea Blvd, Scottsdale, AZ 85259, USA

**Keywords:** Myelofibrosis, Targeted therapy, Hedgehog pathway inhibitors, Janus kinase inhibitors

## Abstract

Treatment of myelofibrosis (MF), a BCR-ABL–negative myeloproliferative neoplasm, is challenging. The only current potentially curative option, allogeneic hematopoietic stem cell transplant, is recommended for few patients. The remaining patients are treated with palliative therapies to manage MF-related anemia and splenomegaly. Identification of a mutation in the Janus kinase 2 (*JAK2*) gene (*JAK2* V617F) in more than half of all patients with MF has prompted the discovery and clinical development of inhibitors that target JAK2. Although treatment with JAK2 inhibitors has been shown to improve symptom response and quality of life in patients with MF, these drugs do not alter the underlying disease; therefore, novel therapies are needed. The hedgehog (Hh) signaling pathway has been shown to play a role in normal hematopoiesis and in the tumorigenesis of hematologic malignancies. Moreover, inhibitors of the Hh pathway have been shown to inhibit growth and self-renewal capacity in preclinical models of MF. In a mouse model of MF, combined inhibition of the Hh and JAK pathways reduced *JAK2* mutant allele burden, reduced bone marrow fibrosis, and reduced white blood cell and platelet counts. Preliminary clinical data also suggest that inhibition of the Hh pathway, alone or in combination with JAK2 inhibition, may enable disease modification in patients with MF. Future studies, including one combining the Hh pathway inhibitor sonidegib and the JAK2 inhibitor ruxolitinib, are underway in patients with MF and will inform whether this combination approach can lead to true disease modification.

## Myelofibrosis

Myelofibrosis (MF) is one of several BCR-ABL–negative myeloproliferative neoplasms (MPNs), which are derived from multipotent, hematopoietic myeloid progenitors [1,2]. MF can be primary (PMF) or secondary to the MPNs polycythemia vera (PV) or essential thrombocythemia (ET) (post-PV or post-ET, respectively) [2]. MF is characterized by stem cell–derived clonal myeloproliferation, abnormal cytokine expression, bone marrow fibrosis, cytopenias, extramedullary hematopoiesis (eg, splenomegaly), cachexia, and constitutional symptoms including night sweats, fevers, weight loss, and fatigue [3-5]. Disease complications also include infections, portal hypertension, bleeding, extremity pain, and progression of disease with blastic transformation, resembling acute leukemia [5-8].

**Figure 1:**
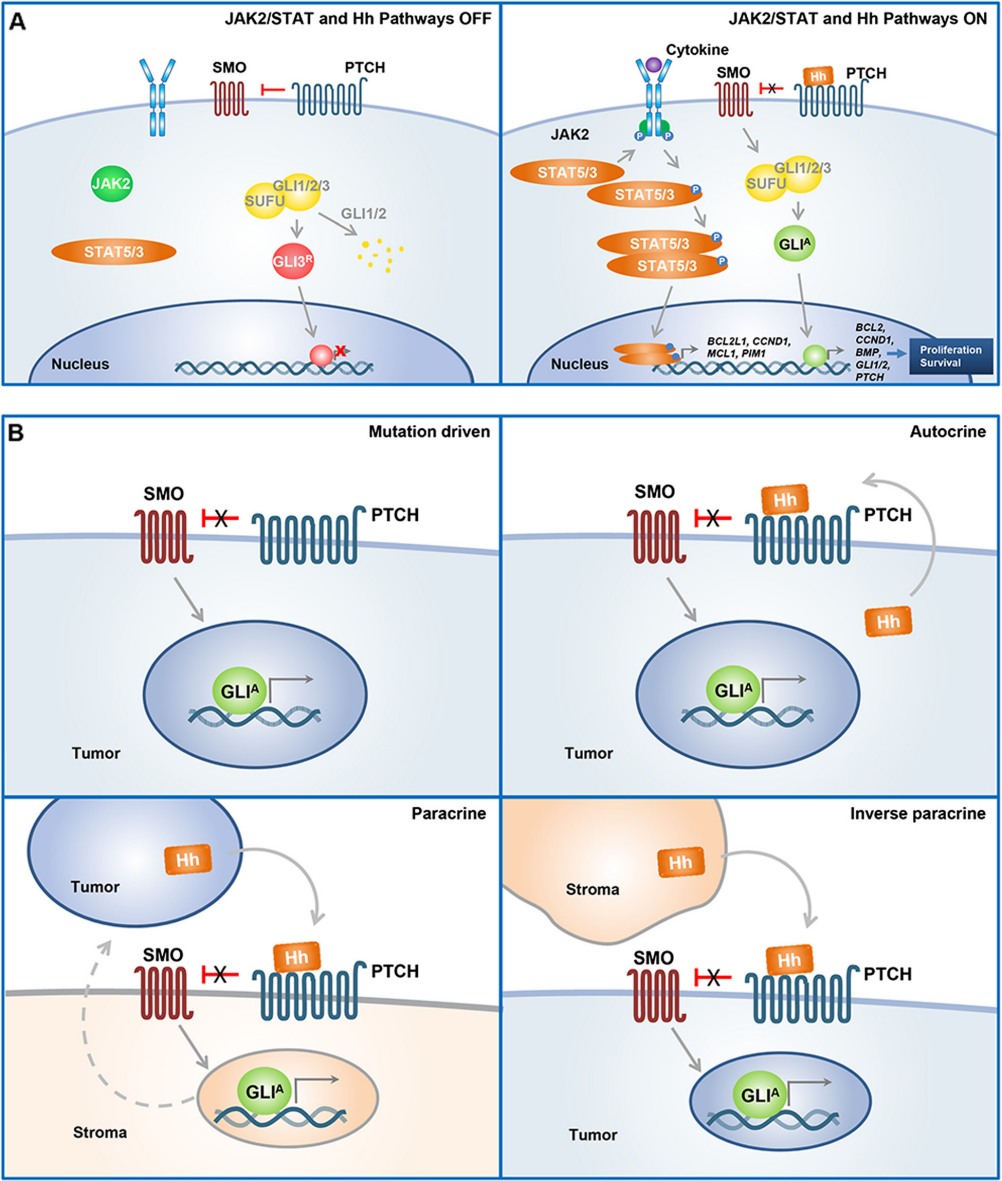
**Janus kinase 2 (JAK2)/signal transducer and activation of transcription (STAT) and hedgehog (Hh) signaling pathways in normal development (A) and mechanisms of Hh signaling in cancer (B). (A)** JAK/STAT signaling: the JAK2/STAT signaling pathway is activated upon binding of a cytokine to its receptor, causing phosphorylation and activation of JAK2, which then recruits and phosphorylates STATs. STATs dimerize, translocate to the nucleus, and activate target gene transcription. Hh signaling: in the absence of Hh ligand, patched (PTCH) inhibits smoothened (SMO). Glioma-associated oncogene homolog 1/2 (GLI1/2) transcription factors are sequestered in the cytoplasm by a repressor complex containing suppressor of fused (SUFU) and degraded. GLI3 is released from SUFU, processed into a repressor form (GLI3^R^), and translocates to the nucleus to inhibit transcription of Hh pathway target genes. Hh signaling is activated upon binding of Hh to PTCH. PTCH-mediated inhibition of SMO is relieved, and SMO activates release of GLIs from the SUFU complex. Activated GLIs (GLI^A^) then translocate to the nucleus to regulate target gene transcription. **(B)** Several mechanisms of Hh pathway activation in cancer have been proposed, including ligand independent (mutation driven) and ligand dependent (autocrine or paracrine) signaling. During autocrine signaling, Hh ligands produced in the tumor activate Hh signaling in the same tumor cells. Paracrine signaling can involve tumor-to-stroma signaling or stroma-to-tumor signaling (reverse paracrine). During tumor-to-stroma signaling, Hh ligands produced in the tumor activate Hh signaling in surrounding stromal cells, which release growth hormones that in turn feed tumor growth. In the reverse model (stroma-to-tumor), which has been observed in hematologic malignancies (lymphoma, myeloid neoplasms, and multiple myeloma), Hh ligands produced in stromal cells activate Hh signaling in the tumor. BCL2, B-cell CLL/lymphoma 2; BCL2L1, BCL2-like 1; BMP, bone morphogenetic protein; CCND1, cyclin D1; MCL1, myeloid cell leukemia sequence 1; PIM1, pim-1 oncogene.

MF is most commonly characterized by a mutation in Janus kinase 2 (*JAK2* V617F), which is present in approximately 96%, 55%, and 65% of patients with PV, ET, and PMF, respectively [5,9]. The *JAK2* V617F gain-of-function mutation leads to constitutive activation of the JAK/signal transducer and activation of transcription pathway, which regulates the expression of genes involved in proliferation, survival, and resistance to apoptosis (B-cell CLL/lymphoma 2-like 1, myeloid cell leukemia sequence 1, pim-1 oncogene, and cyclin D1; Figure 1A) [10]. Recently, a high frequency of calreticulin mutations has been found in JAK2 nonmutated MF [11,12]. Mutations in other genes, including myeloproliferative leukemia virus oncogene, SH2B adaptor protein 3, tet methylcytosine dioxygenase 2, additional sex combs like 1 homolog (ASXL1), isocitrate dehydrogenase (IDH), enhancer of zeste homolog 2 (EZH2), DNA (cytosine-5-)-methyltransferase 3 α, casitas B-lineage lymphoma proto-oncogene, *TP53*, splicing factor 3b subunit 1, and serine/arginine-rich splicing factor 2 (SRSF2), have been found less frequently in patients with MF [5]. Some of these mutations have been linked with poor survival (ASXL1, EZH2, and SRSF2) and/or leukemic transformation (ASXL1, SRSF2, and IDH) in patients with PMF [13].

According to the Dynamic International Prognosis Scoring System Plus (DIPSS Plus), patients with MF are assigned to one of 4 risk groups—low, intermediate-1, intermediate-2, and high. These risk groups are based on 8 factors independently associated with decreased survival: age > 65 years, hemoglobin levels < 10 g/dL, leukocyte count > 25 × 10^9^/L, circulating blood blasts ≥ 1%, constitutional symptoms, red blood cell transfusion, platelet count < 100 × 10^9^/L, and unfavorable karyotype [14]. Median survival varies considerably according to risk group, ranging in one study from 16 to 185 months for high- and low-risk patients, respectively [14].

### Current treatment strategies

The DIPSS Plus and Myeloproliferative Neoplasm Symptom Assessment Form are used to inform treatment regimen decisions [7,15]. For patients with asymptomatic low-risk or intermediate-1 disease, observation is generally recommended [5,16]. For symptomatic patients, current therapies include allogeneic hematopoietic stem cell transplant (HSCT) and palliative treatments that help alleviate disease symptoms such as anemia and splenomegaly [5,16,17]. Allogeneic HSCT is associated with significant risk of morbidity and mortality due to relapse, infection, and graft-versus-host disease, and therefore is recommended only for patients aged < 65 years with intermediate- or high-risk disease [18]. Reduced-intensity conditioning regimens have shown more favorable outcomes but still pose a high risk for patients aged > 55 years and patients with mismatched donors [19].

Therapies intended to treat MF-associated anemia include growth factors (eg, erythropoietin), androgens (eg, danazol), and the immunomodulatory drugs (IMiDs) thalidomide (± prednisone), lenalidomide (± prednisone), and pomalidomide (± prednisone) [20-26]. IMiDs have also been shown to improve splenomegaly [27-29]. Other agents used to treat MF-associated splenomegaly include the nonspecific oral myelosuppressive agent hydroxyurea, the oral alkylators melphalan and busulfan, and the purine nucleoside analog 2-CdA [30-32]. Hydroxyurea is a choice for splenomegaly in patients with MF as well [5]. Although generally well tolerated, hydroxyurea can lead to myelosuppression, which can exacerbate MF-associated anemia [14,16].

Based on the finding that the majority of patients with MF have a mutation in *JAK2*, numerous inhibitors of JAK2 (ruxolitinib [INCB018424], fedratinib [SAR302503; TG101348], lestaurtinib [CEP-701], momelotinib [CYT387], pacritinib [SB1518], AZD1480, BMS-911543, gandotinib [LY2784544], AT9283, and XL019) have been developed and are being evaluated in clinical trials. Of note, JAK inhibitors also have activity in JAK2 nonmutated MF/PMF [33,34]. Ruxolitinib, an inhibitor of JAK1 and JAK2, was approved in 2011 by the US Food and Drug Administration (FDA) for use in patients with intermediate- or high-risk MF (PMF, post-PV MF, and post-ET MF) and in 2012 by Health Canada and the European Medicines Agency for the treatment of MF-related splenomegaly and symptoms [35-37]. JAK2 inhibitors differ according to their specificity for JAK2 and have variable efficacy and toxicity profiles [5,17].

### Unmet need in the treatment of MF

Currently, the only potentially curative therapy for patients with MF is allogeneic HSCT [16,38]. Due to treatment-related morbidity and mortality, HSCT is recommended for patients with intermediate-2– or high-risk disease who are fit enough to undergo the procedure. The majority of patients with MF are treated with palliative therapies, which improve disease symptoms rather than altering the natural history of disease [17]. The discovery of the *JAK2* gain-of-function mutation, *JAK2* V617F [39-42], followed by the development and approval of ruxolitinib has marked a new era in the treatment of MF, providing improved symptomatic responses and quality of life in comparison with traditional therapies [36,37,43-45]. However, treatment with JAK2 inhibitors has shown only limited evidence of disease modification–JAK2 inhibitors do not improve bone marrow fibrosis and most provide limited reduction of *JAK2* V617F allelic burden [16,17]. Ruxolitinib appears to block inflammatory cytokine activity rather than stem cell–derived clonal myeloproliferation, which is the primary driver of the disease [46]. Therefore, disease resistance can ensue following an initial response to JAK2 inhibition [16,46]. In addition, treatment-related anemia may exacerbate preexisting MF-related anemia [33,43,44].

To further improve the responses to JAK2 inhibitors, various combinations have been clinically tested. For example, combination of JAK2 inhibitors with agents that improve anemia (eg, IMiDs) or target signaling pathways involved in proliferation, survival, and self-renewal may further improve the outcome of patients with MF [26,47-49]. Combinations of JAK2 inhibitors with inhibitors of the hedgehog (Hh) pathway, which plays a role in the maintenance of cancer stem cells [50], could provide an avenue of targeting stem cell–derived clonal myeloproliferation (which evades JAK2-targeted monotherapy) [51]. Other combination partners, including hypomethylating agents (Tibes, unpublished observation) and Aurora-kinase inhibtors have also been proposed [52]. The preclinical rationale and current clinical evidence supporting use of Hh pathway–targeted therapies in the treatment of patients with MF will be discussed herein.

## Rationale for targeting the Hh pathway in MF

### The Hh pathway and its role in hematopoiesis

The Hh signaling pathway plays a role in proliferation, differentiation, and survival during embryonic development and in tissue and stem cell maintenance in the adult [50,53]. Hh signaling is initiated when one of 3 ligands–sonic hedgehog (SHH), Indian hedgehog (IHH), or desert hedgehog (DHH)–binds to patched (PTCH), a 12-transmembrane receptor, relieving its inhibition of smoothened (SMO), a 7-transmembrane G-like protein–coupled receptor (Figure 1A). SMO then translocates to the primary cilium and activates the glioma-associated oncogene homolog (GLI) transcription factors, a process that involves their release from a repressor complex including suppressor of fused. Once released, GLIs translocate to the nucleus to regulate the transcription of target genes including *GLI1/2*, *PTCH*, cyclin D1, and B-cell CLL/lymphoma 2.

**Figure 2:**
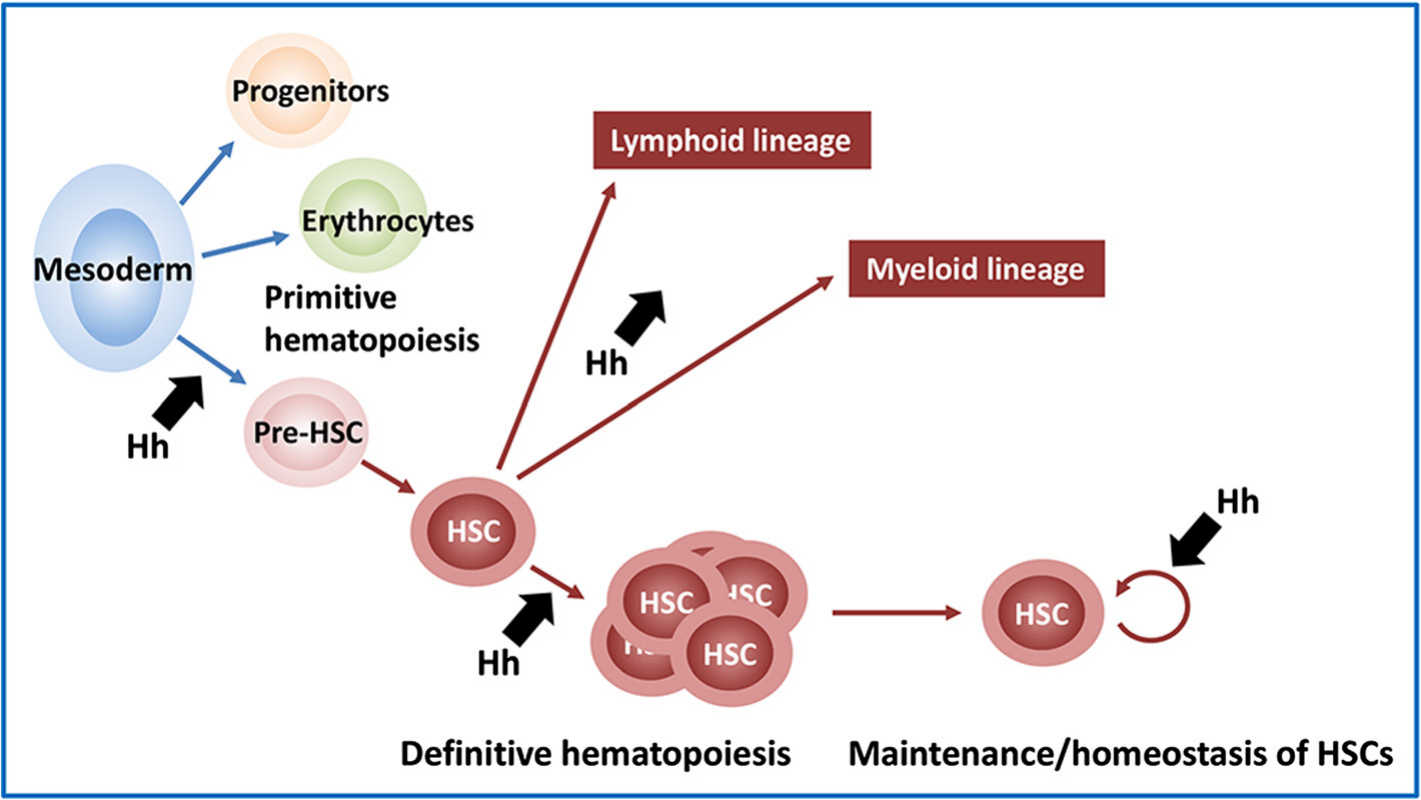
**Role of hedgehog (Hh) signaling in hematopoiesis.** Preclinical studies suggest that the Hh signaling pathway may be involved in numerous stages and processes of hematopoiesis, including primitive hematopoiesis, definitive hematopoiesis—establishment, proliferation, and differentiation (lymphoid and myeloid lineages)—and maintenance of HSCs. The exact role for Hh signaling at each of these stages/processes is not clear. HSC, hematopoietic stem cell.

Hh signaling is required during hematopoiesis (Figure 2); however, its exact role is not completely understood and may differ depending on the stage of hematopoiesis, cell type (stem, primitive, or differentiated cell), and physiological state [54]. During primitive hematopoiesis, when embryonic mesoderm is committed to becoming hematopoietic precursors (eg, erythrocytes) and blood islands begin to form [55,56], *Ihh* is expressed in the visceral endoderm surrounding the epiblast and in the endodermal layer of the mature yolk sac and induces the expression of *Ptch1*, *Smo*, and *Gli1* within these tissues [57]. Murine *Ihh* knockout mice and in vitro studies in *Ihh-*deficient embryonic stem cell lines suggest that Ihh is required for hematopoiesis and vasculogenesis [57-60]. Survival of half of *Ihh*^−/−^ mice and the observation that *Smo*^−/−^ mice die earlier suggest that *Dhh* and/or *Shh* may also play a role in primitive hematopoiesis and vasculogenesis [57,61].

Preclinical studies also suggest that Hh plays a role not only in establishing definitive hematopoiesis, which is characterized by formation of multipotent hematopoietic stem cells (HSCs), but also in the proliferation and differentiation of HSCs (Table 1) [62-70]. Activated Hh signaling through loss of *Ptch* leads to increased HSC formation and activity [64,66], enhanced recoverability following treatment with 5-fluorouracil [65,66], and increased regeneration capacity [65,66]. Conversely, loss of pathway activity through mutation of the downstream effector, *Gli1*, in mice leads to decreased proliferation of long-term HSCs and myeloid progenitors, reduced myeloid differentiation, and delayed recovery following 5-fluorouracil treatment [69]. Interestingly, reduced HSC activity (through loss of *Gli1*) led to increased engraftment. Together, these studies suggest that inhibition of the Hh pathway at different nodes (ie, Smo vs Gli1) affects hematopoiesis differently.

**Table 1:** Preclinical evidence for Hh pathway involvement in hematopoiesis and in progenitor expansion and maintenance

	**Preclinical model**	**Evidence**
**Activated Hh signaling**	*• Ptch* mutant chimeric explant cultures	*•* Hh signaling from ventral tissues surrounding the AGM was shown to induce and increase HSC activity in a time-dependent manner [64]
	*• Ptch*^+/−^ mice vs wild-type mice (bone marrow, fetal liver cells)	*•* Hh signaling enhanced regeneration potential in short-term HSCs through increased HSC number [66], enhanced recoverability following 5-FU treatment [65,66], and increased regeneration capacity [65,66]
		*•* In long-term HSCs with activated Hh signaling, repopulating cells were eventually exhausted in the bone marrow [66], whereas fetal liver cells showed long-term regeneration capacity [65]
	*•* Conditional loss of *Ptch* in adult murine HSCs	*• Ptch* deletion in HSCs did not cause Hh pathway activation or affect hematopoiesis [67]
	*•* Conditional loss of *Ptch* in adult murine non-HSCs	
		*• Ptch* deletion in non-HSCs led to aberrant hematopoiesis, including apoptosis of lymphoid progenitors in epithelial cells, increased numbers of lineage-negative bone marrow cells, and increased mobilization of myeloid progenitors in bone marrow niche cells [67]
**Impaired Hh signaling**	*• Ihh*^−/−^ knockout mice	*•* Terminal erythroid differentiation was defective despite normal production of HSCs and progenitor cells [62]
	*• Dhh-*deficient mouse model	*• Dhh* was shown to regulate normal and stress-induced erythropoiesis by preventing erythropoiesis differentiation in the spleen and bone marrow [71]
	*•* Conditional deletion of *Smo* in fetal and adult hematopoietic and endothelial cells (*Vav* driven *Cre-Lox* system)	*•* Decreased stem cell activity was observed despite normal number and differentiation of HSCs [68]
	*• Smo-*depleted mouse stromal cells	*•* Differentiation of hematopoietic progenitors was impaired—the number of myeloid progenitors was increased at the expense of lymphoid progenitors [72]
		*•* Caused reduced expression of factors involved in B-cell development or osteoblast differentiation
	*• Gli1*^*null*^ mice	*•* Decreased proliferation of long-term HSCs and myeloid progenitors, reduced myeloid differentiation, and delayed recovery following 5-FU treatment were observed [69]
	*•* Human pluripotent stem cells	*• Gli3*^*R*^, the repressor form of *Gli3*, was shown to be necessary and sufficient in the initiation and regulation of adult hematopoietic specification [70]

*5-FU* 5-fluorouracil, *AGM* aorta-gonad-mesonephros, *Dhh* desert hedgehog, *Gli* glioma-associated oncogene, *Hh* hedgehog, *HSC* hematopoietic stem cell, *Ihh* Indian hedgehog, *Ptch*, patched, *Smo* smoothened.

The role of Hh signaling in long-term HSCs is not well understood—several groups have reported conflicting results (Table 1); however, in each study, activated Hh signaling led to aberrant hematopoiesis [65-67]. There have also been some discrepancies in studies involving deletion of *Smo*, based on the temporal expression pattern of the experimental driver used (embryogenesis vs adulthood) and its specificity (hematopoietic and endothelial tissue vs HSCs, lymphocytes, and liver cells) [65,68,73,74]. Disruption of Hh signaling earlier and in more tissues affected HSC function, whereas disruption of Hh signaling in adult HSCs had no effect, suggesting that Hh signaling may be important during early definitive hematopoiesis.

Numerous studies have also presented evidence implicating the Hh pathway in the maintenance or homeostasis of hematopoietic precursors [72,75-79]. Activated Hh signaling in nonhematopoietic cells (ie, epithelial cells or marrow niche cells) led to apoptosis of lymphoid progenitors or an increase in the number of lineage-negative bone marrow cells and increased mobilization of myeloid progenitors [67]. Inhibition of Hh signaling in marrow stromal cells led to impaired differentiation of B-lymphoid cells from hematopoietic progenitors—the number of myeloid progenitors was increased at the expense of lymphoid progenitors [72]. These and several other studies suggest that Hh signaling may be required in a noncell autonomous manner where Hh signaling functions in the nonhematopoietic bone marrow cells (ie, stroma or epithelial cells) surrounding HSCs to maintain, particularly myeloid, hematopoietic precursors (Figure 2) [67,72,76-79].

### The Hh pathway in MF and other hematologic malignancies

To date, preclinical data on the potential role of the Hh pathway in MF are limited. However, in one study, expression of *GLI1* and *PTCH1* were shown to be increased up to 100-fold in granulocytes isolated from patients with MPNs compared with control granulocytes [51]. The Hh pathway was also shown to be up-regulated in a mouse bone marrow transplant model [51]. In this same model, mice were treated with vehicle, ruxolitinib, or a combination of ruxolitinib and the SMO inhibitor sonidegib (LDE225), for 28 days [51]. Combination therapy resulted in increased efficacy in MPNs—causing a greater reduction of mutant allele burden in the bone marrow, reduced bone marrow fibrosis, lower white blood cell count, and lower platelet count than treatment with vehicle or ruxolitinib alone (Table 2). Moreover, in the *Gata1*^*low*^ mouse model of MF, gene expression analysis of the spleen and bone marrow identified alterations in the expression of bone morphogenetic protein 4, an indirect target of the Hh pathway, further supporting a role for Hh signaling in MF [80,81].

**Table 2:** Pharmacologic inhibition of SMO in MF, leukemia, lymphoma, and MM preclinical models and cell lines

**Model**	**Inhibitor**	**Effect**
Murine MF model [51]	Sonidegib (LDE225)	*•* Caused reduction of mutant allele burden in bone marrow, reduced bone marrow fibrosis, and reduced white blood cell and platelet counts when combined with ruxolitinib in comparison with ruxolitinib treatment alone
Murine CML models [65,68]	Cyclopamine	*•* Reduced LSC numbers and secondary transplantation capacity in BCR-ABL+ cells
		*•* Prolonged survival in treated mice, alone [68] or in combination with TKI therapy [65]
BCR-ABL+ cells [82-85]	Vismodegib (GDC-0449), sonidegib	*•* Inhibited cell growth, self-renewal, and serial transplantation
		*•* Enhanced activity of BCR-ABL–targeted TKIs
		*•* Enhanced control of TKI-resistant cells [83,84]
AML cell lines and primary cells [86]	PF-04449913	*•* Inhibited proliferation and induced cell death (minimally)
		*•* Attenuated leukemia initiation potential in serial transplantation experiments
ALL cell lines [87,88,90]	Cyclopamine, saridegib (IPI-926)	*•* Decreased self-renewal as evidenced by decreased numbers of ALDH+ cells; significantly decreased secondary colony formation in vitro and leukemic engraftment in vivo [87]
	Vismodegib	*•* Induced apoptotic cell death (reduced levels of p53 and cyclin D1) [90]
	Sonidegib, BMS-833923	*•* Proliferation and apoptosis were not affected; data support hypothesis that Hh signaling may affect self-renewal [88]
MM CD138− tumor stem cells [91]	Cyclopamine	*•* Significantly inhibited cell growth relative to control

*ALDH* aldehyde dehydrogenase, *ALL* acute lymphocytic leukemia, *AML* acute myeloid leukemia, *CML* chronic myeloid leukemia, *LSC* leukemic stem cell, *MF* myelofibrosis, *MM* multiple myeloma, *SMO* smoothened, *TKI* tyrosine kinase inhibitor.

There are many preclinical studies implicating the Hh pathway in the pathogenesis of other hematologic malignancies and solid tumors [92]. Aberrant Hh signaling in cancer is postulated to occur through ligand-independent and ligand-dependent mechanisms (Figure 1B) [93]. Ligand-independent or mutation-driven signaling occurs when mutations in Hh pathway components—loss-of-function mutations in the negative regulators *PTCH* and *SUFU* (suppressor of fused)*,* or gain-of-function mutations in the positive regulator *SMO—*lead to constitutive pathway activation within tumor cells. This type of signaling has been observed in basal cell carcinoma (*PTCH* and *SMO* mutations) [94,95], medulloblastoma (*PTCH* and *SUFU* mutations) [96], and rhabdomyosarcoma (*PTCH* and *SUFU* loss of heterozygosity) [97].

Ligand-dependent mechanisms involve autocrine or paracrine Hh signaling [93]. During autocrine Hh signaling, tumor cells both secrete and respond to Hh—this type of Hh signaling has been identified in chronic myeloid leukemia (CML), small cell lung cancer, pancreatic cancer, breast cancer, and glioma [93]. Paracrine Hh signaling involves tumor-to-stroma or stroma-to-tumor (reverse paracrine) signaling. During tumor-to-stroma paracrine signaling, tumor cells produce and secrete Hh ligands which activate Hh signaling in surrounding stromal cells. Activated stromal cells release growth hormones which in turn stimulate tumor cell proliferation. Evidence for tumor-to-stroma paracrine signaling has been observed in pancreatic, colon, and prostate cancers [93]. Evidence for reverse paracrine signaling (stroma-to-tumor) in which Hh ligand produced in bone marrow stromal cells activates Hh signaling in surrounding tumor cells, has been reported for hematologic malignancies such as lymphoma, myeloid neoplasms, and multiple myeloma (MM) [91,98]. In addition, the Hh pathway has been implicated in the maintenance and differentiation of cancer stem cells in CML, B-cell acute lymphocytic leukemia (B-ALL), and MM [50,99,100]. Moreover, up-regulation of Hh pathway components has been observed in the tumor stem cells of numerous hematologic malignancies, including BCR-ABL+ leukemic stem cells (LSCs) [65,68], clonogenic B-ALL cells [87], CD34+ acute myeloid leukemia (AML)– and myelodysplastic syndromes (MDS)–derived cells [77], and MM CD138− tumor stem cells [91]. Pharmacologic inhibition of SMO has been shown to inhibit leukemogenesis through inhibition of LSC cell growth, self-renewal, and secondary transplantation capacity and induction of cell death in CML, AML, and ALL models (Table 2) [65,68,82-88]. Hh signaling has also been implicated in the progression of CML in mouse bone marrow transplant models [65,68]. Constitutively active Smo was shown to increase the frequency of CML stem cells and accelerate disease progression [68]. Conversely, genetic loss or pharmacologic inhibition of Smo significantly impaired CML progression and prolonged survival [65,68]. These data suggest that the Hh signaling pathway plays a role in numerous hematologic malignancies, including MF, and its inhibition may block tumor stem cell growth and disease progression.

### Clinical studies of HH pathway inhibitors in patients with MF and other hematologic malignancies

Several Hh pathway inhibitors that target SMO have demonstrated single-agent efficacy in patients with ligand-independent tumors [101-105], including vismodegib, which was approved by the FDA in 2012 for the treatment of patients with locally advanced or metastatic basal cell carcinoma [101,106]. Patients with Hh-activated medulloblastoma have also responded to treatment with vismodegib and the SMO inhibitor sonidegib [102,104,105]. Conversely, limited single-agent activity has been observed in ligand-dependent solid tumors—this lack of activity may be due in part to the contributions of other signaling pathways and stromal factors [107]. To date, saridegib (IPI-926), sonidegib, and PF-04449913 are the only SMO inhibitors that have been or are being tested in patients with MF (NCT01371617, NCT01787552, and NCT00953758, respectively) (Table 3). A phase 2 study of saridegib in patients with MF (NCT01371617) was halted following evaluation of an initial cohort of 12 patients—the level of clinical activity observed with saridegib did not meet the prespecified expansion criteria [108]. No further data have been reported. Data from a phase 1 trial of single-agent PF-04449913 presented at the American Society of Hematology in 2011 showed that PF-04449913 demonstrated activity in patients with refractory, resistant, or intolerant select hematologic malignancies, including MF (NCT00953758) [109]. The dose-limiting toxicity at 80 mg once daily was grade 3 hypoxia and pleural effusion. Of 6 patients with MF treated with PF-04449913, 5 achieved stable disease and 1 achieved clinical improvement with > 50% reduction in extramedullary disease. This patient remained on the study after 385 days and showed a spleen reduction from 10 to 3.5 cm over 8 weeks. Another patient achieved a marked reduction in bone marrow fibrosis.

**Table 3:** Clinical trials of SMO inhibitors in MF and other hematologic malignancies

**Smo inhibitor**	**Patient population**	**Phase**	**Combination partner**	**Primary endpoint**	**Status**^**a**^	**ClinicalTrials.gov identifier**
Sonidegib (LDE225)	PMF, post-PV MF, post-ET MF	1/2	Ruxolitinib	DLTs, MTD and/or RP2D (of combination), proportion of patients achieving ≥ 35% decrease in spleen volume	Recruiting	NCT01787552
	Acute leukemias	2		CR, CRi	Recruiting	NCT01826214
	CML	1	Nilotinib	DLT, MTD, RP2D	Recruiting	NCT01456676
PF-04449913	Refractory, resistant, or intolerant select hematologic malignancies	1		DLT	Completed	NCT00953758 [109]
	AML/MDS	1/2	Chemotherapy	DLT, CRR, OS	Recruiting	NCT01546038
	Acute leukemias	2	Post–stem cell transplant	Relapse-free survival	Recruiting	NCT01841333
	MDS	2		ORR	Recruiting	NCT01842646
Vismodegib (GDC-0449)	AML/MDS	1b/2		ORR	Recruiting	NCT01880437
	Lymphomas (B cell, CLL)	2		ORR	Recruiting	NCT01944943
	MM	1	Post–stem cell transplant	Change in MM CSC counts	Ongoing, not recruiting	NCT01330173
BMS-833923	CML	1/2	Dasatinib	RP2D for combination	Completed	NCT01218477
		2	Dasatinib	MMR	Ongoing, not recruiting	NCT01357655 [89]
	MM	1	Lenalidomide + dexamethasone or bortezomib	DLT, MTD, RP2D	Completed	NCT00884546 [110]

*AML* acute myeloid leukemia, *CLL* chronic lymphocytic leukemia, *CML* chronic myeloid leukemia, *CR* complete remission, *CRi* complete remission with incomplete blood count recovery, *CRR* complete response rate, *CSC* cancer stem cell, *DLT* dose-limiting toxicity, *ET* essential thrombocythemia, *MDS* myelodysplastic syndromes, *MF* myelofibrosis, *MM* multiple myeloma, *MMR* major molecular response, *MTD* maximum tolerated dose, *ORR* overall response rate, *OS* overall survival, *PMF* primary myelofibrosis, *PV* polycythemia vera, *RP2D* recommended phase 2 dose, *SMO* smoothened.

^a^Study status accessed on November 26, 2013 from ClinicalTrials.gov (http://www.clinicaltrials.gov).

Sonidegib is currently being investigated in combination with ruxolitinib in patients with MF in a phase 1/2 study (NCT01787552). Patients with PMF, post-PV MF, or post-ET MF are eligible. Primary endpoints include determination of dose-limiting toxicities, maximum tolerated dose and/or recommended phase 2 dose of the combination, and proportion of patients achieving ≥ 35% decrease in spleen volume. Secondary endpoints include safety, pharmacokinetics, improvement in bone marrow fibrosis, and change in total symptom score (modified Myelofibrosis Symptom Assessment Form v 2.0), *JAK2* V617F allele burden, cytokine levels, and European Organisation for Research and Treatment of Cancer Quality of Life Questionnaire 30.

These inhibitors, as well as the SMO inhibitors vismodegib (first in class) and BMS-833923, are being investigated in other hematologic malignancies, including ALL, AML/MDS, CML, and MM (Table 3) [111].

### Assessment of Hh pathway inhibition in the clinic

For maximization of the potential of Hh pathway inhibitor therapy in patients with MF and related myeloid malignancies such as MDS and AML, and demonstration of a benefit over current therapies, it will be important to develop a method to assess the association of Hh pathway inhibitor activity with efficacy. In other tumor types, GLI1 expression has been used to determine changes in Hh pathway activity and confirm targeted inhibition in patients treated with SMO inhibitors [99,103,104,112,113]. In patients with MF, AML, or CML, one study showed that gene expression analysis of bone marrow CD34+ LSCs before and after treatment with PF-04449913 showed up-regulation of growth arrest specific 1 and kinesin family member 27, 2 negative regulators of the Hh signaling pathway [113]. Although changes in the expression of downstream Hh pathway components can be used to detect Hh pathway repression, a more appropriate measure of Hh pathway inhibitor activity in patients with MF is evidence of disease modification through histopathologic (bone marrow fibrosis) and cytogenetic (*JAK2* V617F allele burden) remission. In patients with MF with *JAK2* V617F mutations, change in allele burden following treatment with a Hh pathway inhibitor would be an appropriate marker for stem cell inhibition. Similarly, for patients with MDS or AML disease-initiating mutations, reduction in allele burden would indicate a possible on-target effect. In patients without mutations, identification of an appropriate marker is yet to be accomplished. Sustained responses following treatment discontinuation may also reflect disease modification. Ultimately, in order to assess the efficacy of future targeted therapies, a combination of endpoints, including disease-specific histopathologic (ie, reduction of fibrosis) and molecular (ie, allele burden reduction) changes and clinical efficacy (ie, improvement in blood counts), should be considered. Future preclinical studies in *JAK2* V617F–negative MF and correlative data from the ongoing trials of Hh pathway inhibitors in patients with MF may better define the optimal method for determination of efficacy and identification of predictive and pharmacodynamic biomarkers in patients treated with Hh pathway inhibitors.

## Conclusions

Despite recent advances in the treatment of MF, lack of true disease modification following treatment with current therapies warrants the identification of novel therapies. Inhibitors of the Hh signaling pathway, which has been implicated in the maintenance of HSCs, have shown preliminary activity as single agents or in combination with ruxolitinib in preclinical and clinical studies in MF. A clinical study combining the Hh pathway inhibitor sonidegib with the JAK2 inhibitor ruxolitinib in patients with MF is currently underway. In addition, we are currently working on preclinical studies and the development of a clinical trial to test the combination of Hh pathway inhibitors with the hypomethylating agent 5-azacitidine (Tibes, personal communication). These and future studies will test the hypothesis that targeting pathways involved in stem cell maintenance will not only extend the duration of benefit but will also lead to true disease modification in patients with MF treated with JAK2 inhibitors, as well as test their activity in other hematologic malignancies.

## Abbreviations

AML: Acute myeloid leukemia; ASXL1: Additional sex combs like 1 homolog; B-ALL: B-cell acute lymphocytic leukemia; CML: Chronic myeloid leukemia; DHH: Desert hedgehog; DIPSS: Dynamic International Prognosis Scoring System; ET: Essential thrombocythemia; EZH2: Enhancer of zeste homolog 2; GLI: Glioma-associated oncogene homolog; Hh: Hedgehog; HSC: Hematopoietic stem cell; HSCT: Hematopoietic stem cell transplant; IDH: Isocitrate dehydrogenase; IHH: Indian hedgehog; IMiD: Immunomodulatory drug; JAK2: Janus kinase 2; LSC: Leukemic stem cell; MDS: Myelodysplastic syndromes; MF: Myelofibrosis; MM: Multiple myeloma; MPN: Myeloproliferative neoplasm; PMF: Primary myelofibrosis; PTCH: Patched; PV: Polycythemia vera; SHH: Sonic hedgehog; SMO: Smoothened; SRSF2: Serine/arginine-rich splicing factor 2; STAT: Signal transducer and activation of transcription; SUFU: Suppressor of fused.

## Competing interests

RT: Research support for clinical trials from Astex, Merck, Celgene, Novartis, Tetralogic, Epizyme and Seattle Genetics. Novartis funding involves support for a clinical trial of the SMO inhibitor (LDE225) with 5-Azacitidine. RAM: Research support from Incyte, Genentech, Sanofi, Gilead, NS Pharma, Lilly, and Promedior.

## Authors’ contributions

RT and RAM contributed to the literature analysis/interpretation and manuscript writing, edited/revised all drafts, and approved the final version of the manuscript.

## Authors’ information

RT: A physician-scientist conducting early clinical trials with novel molecular-targeted agents in patients with myeloid malignancies, including MDS, AML and MPNs/MF. Performing laboratory research to develop new rational therapeutic combinations in acute and chronic leukemias and MF. Involved in early stages of the development of several SMO (Hedgehog pathway) inhibitors including the first-in-class agent vismodegib.

RAM: An accomplished investigator leading clinical developmental efforts and large trials for new agents and therapies in MPNs and MF. Involved in pivotal trials for JAK2 inhibitors.
